# Midterm follow-up of transthoracic device closure of an atrial septal defect using the very large domestic occluder (44–48 mm), a single Chinese cardiac center experience

**DOI:** 10.1186/s13019-017-0639-8

**Published:** 2017-09-02

**Authors:** Qiang Chen, Hua Cao, Gui-Can Zhang, Liang-wan Chen, Fan Xu, Jia-xin Zhang

**Affiliations:** Department of Cardiovascular Surgery, Union Hospital, Fujian Medical University, Xinquan Road 29#, Fuzhou, 350001 People’s Republic of China

**Keywords:** CHD, Septal defects, Cardiac intervention, Transthoracic, Surgery

## Abstract

**Background:**

The purpose of this study was to outline the midterm follow-up results and complications in patients who underwent transthoracic device closure of an atrial septal defect (ASD) with the very large domestic occluder (44–48 mm).

**Methods:**

The data of 35 patients who underwent transthoracic device closure of an ASD with the very large domestic occluder (44–48 mm) at our institution were collected prospectively between January 2010 and January 2015. All patients were invited for an outpatient visit and contrast TTE for 12–70 months after ASD closure.

**Results:**

Thirty-four patients were occluded successfully under this approach and 1 patient was transferred for surgical repair for dislodgement of the occluder. The most frequent complication was transient cardiac arrhythmia. A new third degree atrioventricular block occurred in 1 patient who recovered 1 week later. During the follow-up period, we found no recurrence, no thrombosis, no device embolization, no device failure, and no cases of death. The total occlusion rate was 94.1% in the 12 months of follow-up, and the intracardiac structure and cardiac function were significant improved contemporaneously.

**Conclusion:**

Transthoracic device closure of an atrial septal defect with the very large domestic occluder (44–48 mm) is a safe and feasible technique. However, long-term follow-up is required to better assess the safety and feasibility of this method for the closure of very large ASDs in patients.

## Background

Atrial septal defects (ASDs) account for 10% of congenital cardiovascular malformations of all congenital heart diseases (CHDs) [1]. There are many patients with very large ASDs in clinical practice. In this part of the patient, surgical closure can be achieved with no mortality and minimal morbidity, although this procedure also has some disadvantages [2–4]. In recent years, transcatheter closure with the Amplatzer septal defect occluder has gradually become an alternative treatment for most ASDs [5]. However, a defect that stretches to dimensions of 25 mm or more is relatively difficult to close with the device, and it can be technically challenging even with the device [6]. In recent years, transthoracic device closure for an ASD has been widely used in China. Similar to the other two methods, this method also achieved high technical success and good acute outcomes [7–10]. A small number of reports are accumulating about the immediate and short-term follow-up of transthoracic device ASD closure with the large occluder. However, until now there has been no publication of midterm follow-up results. This report outlined midterm follow-up results and complications in patients with a large ASD that underwent transthoracic device closure using the very large domestic occluder (44–48 mm).

## Methods

The present study was a retrospective study and approved by the ethics committee of Fujian Medical University, China and adhered to the Declaration of Helsinki. Additionally, written informed consent was acquired from the patients or the patient’s relatives.

### Patients

The data from 35 patients who underwent transthoracic device closure using the very large domestic occluder at our institution were collected prospectively between January 2010 and January 2015. All the clinical data are shown in Table 1. All patients underwent pre-operative transthoracic echocardiography (TTE) to confirm the diagnosis of the ASD and to assess the circumferential margins of the ASD. Routine examinations were performed, which included a standard lead electrocardiogram, a chest X-ray examination, and routine blood and biochemical tests. Arrhythmia, sinus bradycardia, and atrial fibrillation occurred in 6 patients before the operation. The chest X-ray showed pulmonary congestion in all patients. All of the patients were symptomatic, with symptoms including palpitation, shortness of breath, exercise intolerance, chest tightness and insignificant chest pain. Of all the patients, 8 had severe pulmonary hypertension (which were accessed by TTE and pulmonary artery systolic pressure were 70–80 mmHg), which still had left to right shunts, and their pulmonary vascular resistance were less than 4 woods units. The rest of the patients had mild-moderate pulmonary hypertension. The inclusion criteria included right atrial and ventricular enlargement, significant left to right shunts, and (or) history of infective endocarditis. Sixteen patients had secundum ASD with the presence of adequate rims (≥5 mm), and the rest of the patients had secundum ASD with a superior/inferior vena cava rim deficiency. The exclusion criteria, similar to what other articles reported, included elevated non-reactive pulmonary vascular resistance (>4 woods), association with other congenital heart disease needing surgical intervention and cardiac function meeting criteria for NYHA III-IV despite medical therapy [11].

**Table 1:** Clinical data of patients undergoing transthoracic device closure of the ASD

Item	
Sex (M:F)	14:21
Age(years)	31.6 ± 8.5
Weight(kg)	52.3 ± 9.5
ASD diameter(mm)	39.1 ± 2.3
Occluder size(mm)	46.2 ± 1.1
Operative time(minutes)	36.2 ± 7.6
ICU stay(hours)	15.3 ± 13.5
Hospital stay(days)	6.5 ± 2.2
Follow-up(months)	34.5 ± 11.4

**Fig. 1:**
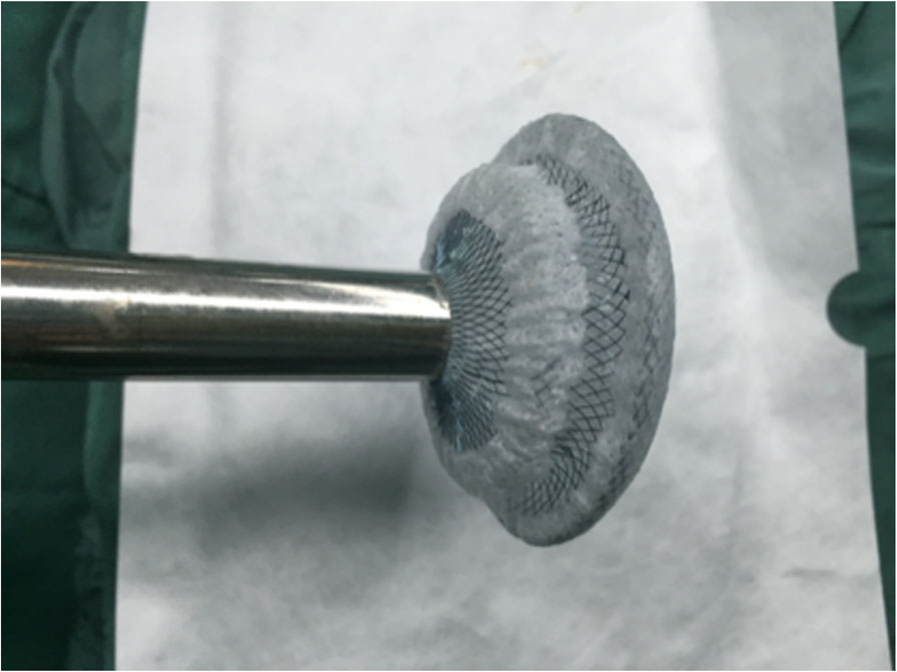
The occlusion device

**Fig. 2:**
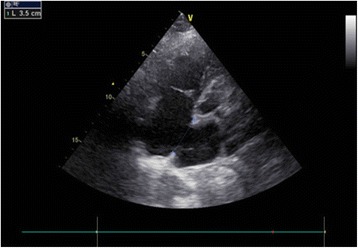
A 35 mm atrial septal defect (ASD)

**Fig. 3:**
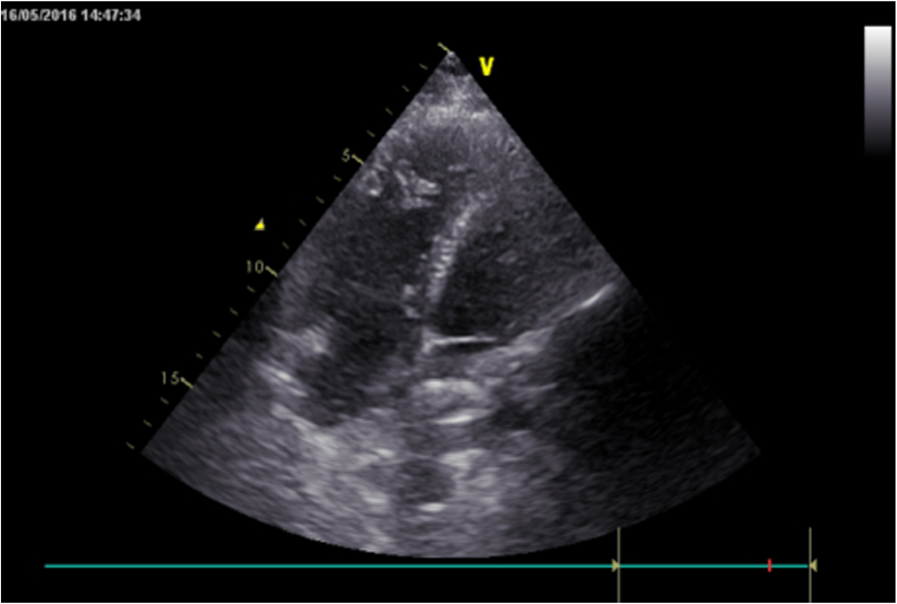
The sheath positioned from the right atrial free wall into the left atrial cavity across the ASD

**Fig. 4:**
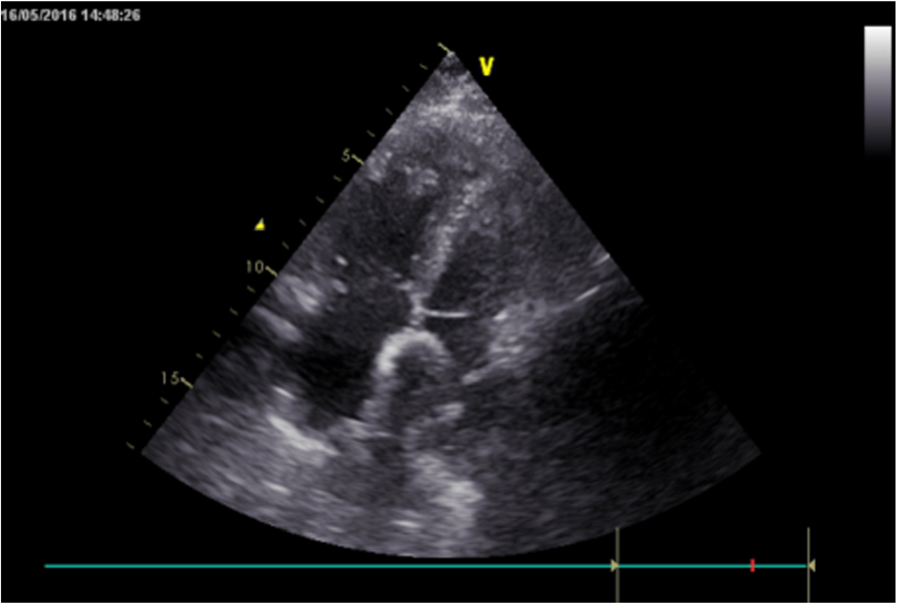
The left disc of an occluder was deployed in the left atrial cavity

**Fig. 5:**
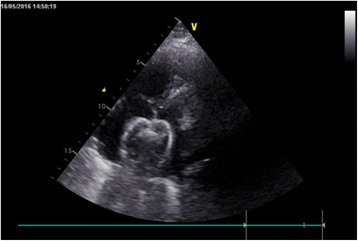
Final image shown after both discs were deployed

The ASD occluder was made in the Dong Guan Ke Wei Medical Apparatus Co. Ltd. and the Shan Dong Visee Medical Apparatus Co. Ltd. of China, which was similar to the Amplatzer ASD occluder [Fig. 1]. In our previous published reports, we had introduced this domestic device and the operating procedures [10]. The device consists of an occluder made from an alloy of nickel and titanium, a sheath, a pushing rod. The right disc with a thread through the disc, facilitating its withdrawal into the sheath. (which was the significant different from the Amplatzar Atrial Septal Occluder) A right anterior sub-mammary mini-thoracotomy (about 5 cm in length) was made through the fourth intercostal space. Through this incision, a “purse-string” suture of approximately 20 mm in diameter were stitched in the right atrium. Heparin was intravenously given (1 mg/kg body weight) before the procedure. The occluder was drawn into the delivery sheath, and then a incision was opened in the “purse-string” suture and the delivery sheath was inserted into the right atrium. Under continuous TTE guidance, the sheath was advanced through the ASD into the left atrium. Then the left and the right disc was deployed in turn by pushing the rod to closure the ASD. [Figs. 2, 3, 4, 5] After the operation, aspirin (100 mg/d) was administered for about 3–6 months.

The diameter of the ASD was measured by TTE, using two-dimensional imaging and colour flow Doppler on apical four chamber and subxiphoid views. In the perioperative and follow-up period, echocardiography was used to assess the sizes of the left and right atria and ventricles, right ventricular fractional area change, right ventricular Tei index and left ventricular ejection fractions, and mitral and aortic regurgitation. Valve regurgitation was evaluated by colour Doppler flow imaging in the standard way [12, 13]. A residual shunt was considered to be present when colour Doppler flow mapping showed a left-to-right shunt across the atrial septum. A shunt was defined as trivial (<1 mm colour jet width), small (1–2 mm), or greater than small (any flow >2 mm) [6].

All patients underwent clinical examination, chest X-rays, and TTE before discharge, at 3 and 12 months after the procedure and yearly thereafter. Patients were followed routinely in an outpatient setting and data were recorded. A platelet anti-aggregation therapy dose of aspirin (100 mg p.o. per day) was administered for 6 months post-operation.

### Statistical analysis

The data were expressed as a frequency or percentage for nominal variables, as the median (range) for categorical variables and as the mean ± SD for continuous variables. Differences in the time periods of data were compared with the analysis of variance with repeated measurement data, using the Tukey HSD test. A *P* value less than 0.05 was defined as statistically significant.

## Results

During the study period, 34 patients were occluded successfully using this approach and 29 of them used the additional suture technique. The diameter of the atrial septal defect ranged from 36 to 42 mm (39.1 ± 2.3 mm), and the size of the implanted occluder ranged from 44 to 48 mm (46.2 ± 1.1 mm). All patients who successfully underwent device closure were monitored in the ICU for 6–24 h and thereafter were transferred to the common ward. The mean total duration of stay in the hospital was 6.8 days.

The comparison between this group of patients and a group of 25 other cases in our division who received conventional surgical repair in the same period (unpublished data) was made. There were no significant differences in the patient choices between these two groups. The results showed that there was a significant difference in the operative time, ICU supervision time, post-operative hospitalization time, the rate of complication and the cost (*P* < 0.05). The device group had a clear advantage, which was the same as the results we had previous reported [10].

The successful ASD closure rate was 82.4% immediately after the operation. Those patients who still had small residual shunts had them in the junction of the occluder and the deficient rim or in the occluder itself. At 3 months and 1 year’s follow-up, only 2 out of 34 patients still had small residual shunts and they had no clinical haemodynamic significance. No new shunts were observed in any patient. One patient was transferred to emergent surgery to retrieve the occluder. This patient had a 38 mm ASD and a 46 mm occluder, and adequate rim was confirmed in the preoperative preparation. After the incision was closed, the occluder was dislodged. Then, a thoracoscope assisted patch closure was selected as a remedial measure.

One patient with a 40 mm ASD and a 44 mm occluder developed post-procedure third degree atrioventricular block (AVB). As heart rates ranged from approximately 50–55 bpm, no pacemaker was needed. After being treated with a glucocorticoid for 1 week, the AVB resolved spontaneously. There were no other serious complications (including cerebral embolism, cardiac perforation, endocarditis, or atrioventricular valve distortion) or mortality in this study. The incidence of minor complications was also acceptable, which included transient cardiac arrhythmia in the course of the device deployment, surgical wound complications, pericardial effusion or hydrothorax. Temporary atrial premature beats, sinus bradycardia and tachycardia were observed in 15 patients, none of which were intervened on and were just observed closely. Three patients who suffered from surgical wound complications, including fat liquefaction of the incision, had their incision sutured again. A large amount of pericardial effusion or hydrothorax occurred in another 2 patients, requiring only medical drainage tube placement. Two patients suffered from pulmonary infections post-operation and needed antibiotic treatment.

Follow-up data were obtained from the 34 patients who successfully underwent device closure. No patients were lost to follow-up. The median duration of follow-up was 35 months (range: 12–70 months). There were no late occluder embolizations or surgical interventions. No patient had newly emerging cardiac symptoms or abnormal physical signs except those consistent with preoperative morbidities. No deaths, thromboembolic events, other major complications, or progressive moderate or severe pulmonary hypertension were identified in the follow-up period. Post-operation, there were three new jets of mild tricuspid regurgitation, but no new mitral regurgitation. The occluder was not in contact with the mitral and tricuspid valve. No patient had evidence of aortic regurgitation or erosion. To date, none of the patients developed complete heart block.

Table 2 shows changes in the size and the function of the heart before and after ASD closure with the large domestic occluder (part of the cases who received full 3 years’ follow-up). At 3 months, 1 year’s and 3 years’ follow-up after ASD closure, the end-systolic length and width of the right atrium, length and width of the right ventricle, the right ventricular fractional area change, and right ventricular Tei index were significantly reduced compared with the preoperative data (*P* < 0.05). Meanwhile, the end-systolic length and width of the left atrium, the width and length of the left ventricle and the left ventricular ejection fraction had increased significantly (*P* < 0.05).

**Table 2:** Changes in heart size before and after atrial septal defect closure

Item	Pre-operation	3 months after procedure	1 year after procedure	3 years after procedure
End-systolic length of the right atrium (mm)	75.6 ± 9.5	54.4 ± 6.9*	51.3 ± 7.2*	50.8 ± 6.9*
End-systolic width of the right atrium (mm)	60.2 ± 10.3	49.3 ± 7.5*	44.6 ± 8.5*	44.4 ± 7.6*
End-diastolic length of the right ventricle (mm)	68.6 ± 10.5	59.2 ± 12.3*	58.3 ± 9.5*	57.8 ± 8.6*
End-diastolic width of the right ventricle (mm)	55.6 ± 9.4	51.3 ± 8.7	45.7 ± 7.4*	45.3 ± 8.2*
The right ventricular fractional area change	50.1 ± 3.5	44.3 ± 3.2*	42.5 ± 3.1*	42.2 ± 4.2*
RV-Tei	0.49 ± 0.08	0.34 ± 0.02*	0.31 ± 0.04*	0.31 ± 0.05*
End-systolic length of the left atrium (mm)	42.3 ± 4.5	48.4 ± 6.3*	46.5 ± 5.8*	45.7 ± 6.1*
End-systolic width of the left atrium (mm)	33.2 ± 3.5	37.2 ± 4.3*	37.5 ± 4.2*	37.6 ± 4.5*
End-systolic width of the left ventricle (mm)	27.6 ± 4.2	31.5 ± 5.6*	32.6 ± 5.7*	32.2 ± 5.4*
End-systolic length of the left ventricle (mm)	37.7 ± 7.5	46.1 ± 7.8*	51.7 ± 8.6*	51.2 ± 5.2*
Left ventricular ejection fraction (%)	52.2 ± 5.1	60.4 ± 4.6*	62.1 ± 5.3*	61.8 ± 4.8*

*Different from Preoperative (*P* < 0.05)

## Discussion

The excellent results and very low probability of long-term morbidity following the surgical closure of ASDs with midline sternotomy have been well established in the past years [2–4]. With the development of various devices, transcatheter device closure of ASD also achieve the same clinical result as surgical closure, and contains some advantages, such as no scar, no postoperative pain, no general anaesthesia and a very short hospital stay [14–16]. It still is technically challenging for those with a large ASD, however. Compared with the above two methods, transthoracic device closure of ASDs, as another hybrid minimal invasive technique, can provide a cost-effective, relatively cosmetic and less-invasive results [10]. It also has been proven to have broader indications for device closure of ASDs. In our review of the previous literature, we found that the immediate- and short-term results of this procedure have been sporadically reported, but the midterm follow-up results have not been described. We have described our initial and midterm experience in a single institutional series of 34 patients with ASDs managed with the very large domestic occluder (44–48 mm).

Experience in transcatheter closure of large secundum atrial septal defects is more recent, but most of the large ASD cases of choice were less than 40 mm, which means that it is still a challenge for transcatheter closure of large atrial septal defects [17–19]. Fraisse A. et al. concluded that large ASDs (>38 mm) and defects with deficient rims did not influence the results of their transcatheter device closure, but these patients were referred for surgical closure in their paper [20]. Lopez K reported a series of patients with large secundum atrial septal defects who underwent attempted transcatheter device closure using the larger occluder (maximum diameter of 40 mm) [21]. They emphasized that the failure of the procedure and the device embolization were related to the large size of ASDs. Hongxin L. et al. reported their successful experience with the short- and mid-term results of transthoracic device closure in large secundum ASDs (20–37 mm, and about half of patients with one short rim) [22]. They confirmed that their method was a safe and feasible technique in closing large ASDs. The increased embolization rate of 40 mm devices may be due to anatomical factors (larger defects), which may limit the application of transcatheter device closure in such patients. Our approach was the same as Hongxin L’s and we have more experience with the larger occluder (44–48 mm). With the small improved design, and as the device size and the resistance to dislodgement increases, it could be hypothesized that our method may be suitable for patients with large ASDs.

In our study, 34 (97.1%) patients had successful procedural outcomes. The one patient with an unsuccessful procedural outcome was referred to surgical repair [23, 24]. At first, the 44–48 mm domestic occluder was utilized in a few selected patients in our center who had adequate rims of their defect, using the transthoracic method. This method could provide a perpendicular angle to the atrial septum, which may result in moving the occluder by the shield to the ideal and suitable position and easily deploying the occluder into the defect. At this point, compared with the transcatheter approach, there was a significant advantage in terms of the operation. The other advantage of the technology in our method was the ‘left atrium-occluder-the right atrium’ suture through Waterston’s groove, which can fix the occluder where the transcatheter approach cannot reach. According to the reports in the literature and clinical observation, occluder dislodgement or embolization was a catastrophic complication in the device closure procedure, and the deficient rims were a major cause of the unsuccessful device procedural outcomes [25, 26]. Through such additional stitching, the occluder could be firmly fixed in its position in the case of its dislodgement or embolization. Even for the patients with deficient ASD rims, this method was still feasible and effective. In the case of the failure in this study, we did not use this suture technique, which may lead to the occluder dislodgement. After using the additional stitching technique, there were no more occluder dislodgements or embolizations that occurred in our center.

Atrial tachyarrhythmias, thromboembolism, complete heart block, transient ischaemic attacks, and even sudden death are the other complications reported after device closure, especially large ones [6, 27]. Complete heart block occurred in one patient in our study. This patient maintained stable haemodynamics and resolved spontaneously by using glucocorticoid treatment for about one week. In our follow-up, there were no new AVB onsets. We could hypothesize that the possible mechanism of the AVB was an inflammatory response and subsequent oedema as a correlative result of mechanical rubbing of the occluder against the proximal conduction system. However, the exact mechanism underlying AVB following ASD closure has still remained speculative and controversial. So we recommended abandoning the procedure once complete AVB occurred. Follow-up at one year showed that there was no progression of aortic and/or mitral regurgitation, which is similar to other reports [28, 29].

Although small residual shunts were found in some patients during the occluder release, the position of the shunt was in the junction of the occluder and the deficient rim or in the occluder itself, and they did not need further medical treatment except for close observation. During the follow-up, the shunt wound became trivial or disappeared, due to the formation of endothelialization and the neointima fully closing, covering any residual shunting [30, 31]. In our opinion, as long as the location of the occluder was firm and the left to right shunt decreased significantly, a small residual shunt can be ignored, and the corresponding clinical results were still good for the patients. In fact, the trivial-small residual shunts did not lead to any related complications.

During the follow-up period, all patients had a successful clinical outcome, with no deleterious ECG, echocardiographic or clinical symptom changes. All of the patients treated in this series had a significant left to right shunt and right atrial and right ventricular volume overload. In adults with a very large ASD, volume overload of the right heart system can occur later because of a long-standing shunt. In our study, we provided the complete three years follow-up transthoracic echocardiographic data of part of cases. The postoperative data showed significantly improved heart structure and function compared with the preoperative data, which was mentioned in our previous report and is similar to others’ results [32, 33]. These were mainly because the left to right shunt was blocked, the right heart volume and pressure load rapidly decreased, and the load of left heart system capacity increased correspondingly. In the 3 month, 1 year and 3 years follow-up, remodelling occurred in the expanding right atrium and ventricle. The corresponding changes also occurred in the left atrium and ventricle. As our data showed, RV-FAC and RV-Tei index had reduced and LV-EF had increased, which meant the cardiac function was significantly improved and could reach a new equilibrium after a period of recovery time [32, 33]. These were justified on the grounds that ASD closure with the large device also avoided long term heart dysfunction. Takaya Y and his colleagues evaluated the impact of mitral regurgitation after transcatheter ASD closure. They considered that mitral regurgitation deterioration occurs in a minority of adult patients after transcatheter ASD closure, and may be provoked by geometric changes in the mitral valve annulus, especially in women with advanced age. But, fortunately, our study did not obtain similar results [34].

The large ASD closures might easily cause pulmonary congestion or even oedema, especially in those patients with severe pulmonary hypertension [35]. Bruch L et al. reported their experience on using a fenestrated ASD occluder for a series of 15 patients with advanced age and/or left or right heart failure and/or pulmonary arterial hypertension. Although their results showed that high-risk ASD occlusion can be successful accomplished without any severe complications by a fenestrated occluder, we still insist that it is cautious to choose a device closure of a large ASD for those patients with severe pulmonary hypertension [36]. In our study, those 8 patients with severe pulmonary hypertension were repeatedly evaluated for the possibility of ASD closure. Intravenous treprostinil and/or oral sildenafil or bosentan for 2 weeks before ASD closure, then the echocardiography and the cardiac catheterization were used to measure the pulmonary artery pressure, which was used to decide whether to close the ASD. After device closure, the above drug was continually used in the follow-up for approximately 3 months – 1 year. Echocardiography showed a trend towards a reduction in the pulmonary artery pressure and the improvement of cardiac structure. Symptoms had improved significantly. Meanwhile the exercise capacity was increased, as indicated by the increase in the 6 MWT distance. No progressive severe pulmonary arterial hypertension or heart failure were detected in the follow-up.

Although we are unable to draw any specific indications in regards to patients with such a large ASD, based on our experience with the large occluder, these patients should be approached judiciously, particularly in relation to the individualized anatomic relationship. In our opinion, our method could expand the indications for device closure over the transcatheter method. For most patients, as long as our suture technique can be applied, device closure can be successfully achieved. Even with device closure failure, the occluder embolism cannot reoccur. It can be easy to extend it for conversion to a regular thoracoscope-assisted patch closure without additional incisions, which may ensure the safety of the procedure. So in our opinion, if the largest diameter of the ASD is greater than 30 mm, choosing our method is obviously more sensible. There was some other merit in our method which is stated as follows: its short delivery was easier to advance across the defect and adjust the device to an anchor properly. Secondly, the surgeon can directly push and pull the occluder to check its stability. Third, the device can easily be retrieved through the large sheath.

As in any retrospective study, there was bias associated with data collection and enrolling patients, which were not randomized. Additionally, this was a single institution experience with a small number of patients. Greater experience, possibly in multicentre trials, and long-term follow-up are needed to better assess the safety and feasibility of this procedure. It is possible to establish the “standard treatment” as an alternative to transcatheter and surgical approaches in patients with large ASD.

## Conclusions

In conclusion, our study demonstrated that transthoracic device closure of an atrial septal defect with the very large domestic occluder (44–48 mm) is feasible with an acceptable failure and complication rate. Proper case selection is vital and specific suture technical modifications appear useful for these patients. Although the midterm follow-up result is encouraging, close long-term follow-up is needed to determine the safety and feasibility of this procedure.

## Abbreviations

ASD: Atrial septal defect
AVB: Atrioventricular block
CHD: Congenital heart disease
ICU: Intensive care unit
TTE: Ransthoracic echocardiography
